# Regular postexercise sauna bathing does not improve heart rate variability: A multi‐arm randomized controlled trial

**DOI:** 10.14814/phy2.70449

**Published:** 2025-07-03

**Authors:** Earric Lee, Sascha Ketelhut, Petri Wiklund, Joel Kostensalo, Iiris Kolunsarka, Hans Hägglund, Juha P. Ahtiainen

**Affiliations:** ^1^ Faculty of Sport and Health Sciences University of Jyväskylä Jyväskylä Finland; ^2^ Montreal Heart Institute Montréal Quebec Canada; ^3^ École de kinésiologie et des Sciences de l'Activité Physique Université de Montréal Montréal Quebec Canada; ^4^ Institute of Sport Science, University of Bern Bern Switzerland; ^5^ The Exercise Translational Medicine Center and Shanghai Center for Systems Biomedicine Shanghai Jiao Tong University Shanghai China; ^6^ Natural Resources Institute Finland (Luke) Joensuu Finland; ^7^ Section of Hematology, Department of Medical Sciences Uppsala University Uppsala Sweden; ^8^ Department of Cellular Therapy and Allogeneic Stem Cell Transplantation, Karolinska Comprehensive Cancer Center Karolinska University Hospital Huddinge Stockholm Sweden

**Keywords:** cardiovascular risk factor, exercise training, heart rate variability, heat therapy, sauna bathing

## Abstract

Regular exercise has been shown to increase heart rate variability (HRV) for different populations. Acute and short‐term studies using heat therapy and sauna bathing have also shown HRV improvements. However, long‐term adaptations in HRV to regular exercise and sauna bathing remain unexplored. In this 1:1:1 multi‐arm trial, sedentary participants (*n* = 38) aged 49 ± 9 years with at least one CVD risk factor were randomly assigned to regular exercise and 15‐min postexercise sauna (EXS), regular exercise only (EXE), or control (CON) group, for an 8‐week intervention. Indices of HRV (RR interval, RMSSD, SDNN, resting heart rate [HR], HRMAX–HRMIN, high frequency power [HFP], and low frequency power [LFP]) were measured before (PRE) and after (POST) the trial. Compared to CON, EXE increased the time‐domain measure of HR_MAX_–HR_MIN_ (*p* = 0.003), and elicited significantly smaller decreases in the frequency‐domain measure of LFP (*p* = 0.022). There were no statistically significant differences between EXS and EXE for any of the HRV indices measured. Eight weeks of regular exercise conferred positive changes in both time‐ and frequency‐domain measures of HRV. However, adding regular sauna bathing postexercise offered no additional benefits to HRV over regular exercise alone.

## Graphical abstract

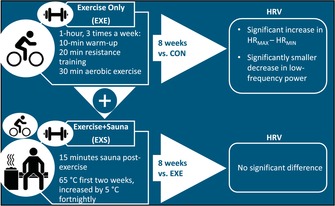

## 1 INTRODUCTION

Elevated blood pressure (BP), a family history of coronary heart disease, high body mass index (BMI), hyperlipidemia, and a smoking background increase the risk for cardiovascular diseases (CVD) (Dahlöf, 2010). Physical activity and exercise training provide protective effects against CVDs (Nocon et al., 2008; Piepoli et al., 2020) and enhance heart rate variability (HRV) (Sandercock et al., 2005), particularly when evidence‐based guidelines are followed (Franklin et al., 2022). However, whether postexercise sauna bathing offers additional HRV improvements over regular exercise remains unexplored.

HRV reflects the variation in the time interval between consecutive heartbeats and is a feasible and noninvasive measure of cardiac autonomic function, predictive of cardiovascular morbidity and mortality (Mccraty & Shaffer, 2015; Shaffer & Ginsberg, 2017). HRV is highly sensitive in detecting subclinical changes in cardiac autonomic function (Castiglioni et al., 2022) and is a measure of both the sympathetic and parasympathetic nervous systems. A higher HRV indicates a healthy and fit cardiovascular system, with a greater capacity to respond to physiological stressors (Kristal‐Boneh et al., 1995). On the other hand, a reduced HRV is associated with diseases and health risks such as hypertension (Singh et al., 1998), sudden cardiac death (La Rovere et al., 2003), and other CVDs (Hillebrand et al., 2013). Therefore, higher HRV reflects responsive parasympathetic activity, whereas lowered HRV indicates unresponsive cardiac autonomic modulation and heightened sympathetic activity, especially in the presence of pathologies (Malik et al., 1996; Mccraty & Shaffer, 2015).

To understand the adaptations of HRV to sauna bathing and exercise, it is essential to first consider their similarities in acute effects, particularly because long‐term studies on sauna bathing are scarce. Evidence suggests that many of the acute physiological responses of exercise and sauna bathing appear to be comparable (Ketelhut & Ketelhut, 2019; Kukkonen‐Harjula et al., 1989; Lee et al., 2021). Indeed, it has been previously reported that acute sauna bathing leads to a concurrent decrease in parasympathetic and an increase in sympathetic activity (Bruce‐Low et al., 2006). However, after 30 min of recovery from sauna bathing, the HRV indices of parasympathetic activity were shown to be significantly higher compared to baseline (Laukkanen et al., 2019), indicating acute cardiac autonomic nervous system balance improvement. In the first study comparing sauna bathing and a combination of exercise and sauna bathing (Gayda et al., 2012), the authors found that a single session of sauna bathing decreased parasympathetic and increased sympathetic control of the heart. However, aerobic exercise followed by sauna bathing did not yield any additional changes in HRV compared to sauna bathing alone.

Long‐term exercise has been reported to increase parasympathetic activity and reduce sympathetic activity, resulting in higher resting HRV (Messina et al., 2012; Routledge et al., 2010). However, this has yet to be investigated extensively in long‐term sauna bathing, or in a combination of long‐term exercise and sauna bathing. Nevertheless, HRV improvements have been reported with 2 weeks of 15‐min sauna sessions performed daily (Kihara et al., 2004); although this involved a clinical population and may not reflect the responses in the general population. We have previously shown that 8 weeks of exercise training in conjunction with sauna bathing confers greater improvements in cardiorespiratory fitness and systolic BP than regular exercise training alone (Lee et al., 2022). These results are encouraging for populations with CVD risk factors, who stand to gain the most from lifestyle interventions (Arnett et al., 2019).

Despite the known benefits of regular exercise on HRV, the potential additive adaptations of postexercise sauna bathing remain underexplored. Using add‐on exploratory data from our original published manuscript (Lee et al., 2022), we aim to fill the gap by examining the changes in HRV from regular exercise, with and without postexercise sauna bathing. We hypothesized that combining sauna bathing with regular exercise may provide additional benefits for improving HRV, especially among populations at risk for CVD.

## 2 METHODS

### 2.1 Study participants

The randomized controlled trial was registered prior to participant enrollment (clinicaltrials.gov; Unique identifier: NCT04540718) in August of 2019, completed in December of 2019, and was conducted in accordance with CONSORT guidelines (Boutron et al., 2017). Adult participants of both sexes (females = 41, males = 6) between 30 and 64 years of age were recruited from the region of Central Finland, Jyväskylä. The inclusion criteria of the study were having at least one traditional CVD risk factor (elevated BP, high cholesterol, family history of coronary heart disease [CHD], obesity, or smoking) and a sedentary lifestyle; defined as having a desk‐bound job and less than 30 min of exercise weekly. Baseline resting systolic BP >139 mmHg and/or diastolic BP >89 mmHg was considered elevated (Williams et al., 2018). Total cholesterol level >6.2 mmol/L was considered high. Family history of CHD was positive if father (<55 years) or mother (<65 years) had premature CHD. BMI >30 kg/m^2^ was defined as obese.

Exclusion criteria were (1) more than once a week of sauna bathing within 6 months of study commencement, (2) commuting to work via physical activities, for example, running, cycling, etc., (3) previously diagnosed CHD and/or diabetes, and (4) any diagnosed and/or symptomatic CVD, musculoskeletal injury, or any other physical or mental condition within 6 months prior to the start of the study. None of the participants were on any medications during the study. The ethical approval was granted by the institutional review board of the Central Finland Hospital District ethical committee, Jyväskylä, Finland (Dnro 3 U/2019). This study was conducted in accordance with the principles of the Declaration of Helsinki, and all participants provided written informed consent prior to enrollment.

### 2.2 Experimental design

A 1:1:1 allocation using simple randomization stratified by biological sex was used to assign participants to either the EXS (exercise and sauna), EXE (exercise only), or the CON (control) group. The randomization sequence was created using Excel 2016 (Microsoft, Redmond, WA) by the corresponding author (EL), and a Masters degree student involved in the project (IK) enrolled the participants. They were then assigned to their respective interventions by a member of the research team who was uninvolved with the data collection and analysis process. The experiment consisted of two measurement days completed by all three groups before (PRE) and after (POST) the study period, and an 8‐week intervention for the EXS and EXE groups. The intervention adhered to the Finnish national guidelines (UKK‐instituutti, 2023), which are based on international guidelines for exercise testing and prescription (Liguori & American College of Sports Medicine, 2020). The exercise sessions were held three times a week in the evenings. They were carried out in groups of 1–5 participants under the supervision of two qualified instructors. Each session lasted an hour and was performed in a specific order, starting with a 10‐min full‐body warm‐up, followed by 20 min of resistance exercise, and concluding with 30 min of aerobic exercise. The adherence rate was approximately 95%, with only two participants missing a session each (Lee et al., 2022).

Aerobic exercise was performed using Monark cycle ergometers (Monark 828 E, Varberg, Sweden). Aerobic exercise intensity began at 65% of individually estimated age‐based maximum heart rate (HR) (Nes et al., 2013), with an increase of 5% every fortnight. HR and pedaling frequency (65–70 revolutions per minute) were monitored by exercise instructors and verified every 5 min. Based on the targeted HR, the resistance of the bike ergometer was adjusted to achieve the specified exercise intensity. The HR monitor was synced and displayed on the ergometer, and the instructors held on to each participant's watches. Participants in the EXS group proceeded immediately to the sauna room after aerobic exercise, while those in the EXE group were instructed to wait in the gym. The temperature of the gym was between 20°C and 22°C at all times, and participants were allowed to consume fluids ad libitum. They were allowed to leave after the EXS group completed their 15‐min sauna exposure. The temperature of the sauna was monitored and recorded every minute. It was set to 65°C for the first 2 weeks and was increased by 5°C fortnightly. Relative humidity of the sauna room was between 10% and 20%. Participants were permitted to leave the sauna at any time, but they all completed 15 min of continuous sauna bathing for every session successfully.

All measurements took place in the exercise and health laboratory (University of Jyväskylä) in the morning between 06:30 and 09:30 in fasted conditions. Participants were instructed to abstain from food, drinks, alcohol, and nicotine for 12 h and to refrain from heavy physical activity for 48 h before all measurements. The measurements were taken 48 h, but not more than 72 h, after the last session.

### 2.3 Measurement of heart rate variability

Participants put on a chest strap and HR monitor (Polar V800; Polar Electro Oy, Kempele, Finland) before proceeding to lie down in a supine position for 5 min of bed rest. Thereafter, HRV data was acquired for an uninterrupted period of 8 min. During the measurement, participants were instructed to avoid sleeping, talking, and any unnecessary movements. All measurements took place in a quiet, dimly lit room with a stable temperature (21°C). Respiration was not controlled for during the time of the measurement. The validity of the Polar V800 and its ability to derive HRV variables and produce RR interval recordings consistent with an ECG from these recordings have been documented previously (Giles et al., 2016). Raw recorded data were analyzed by Kubios HRV Scientific (version 4.1.0), which is advanced software for HRV analysis. The software computes all the commonly used time‐domain and frequency‐domain HRV and several nonlinear variables based on beat‐to‐beat RR interval data (Tarvainen et al., 2014).

To ensure comparability and consistency, the recordings used for data analysis were standardized. A fixed 5‐min segment, starting from the 2‐min mark to the 7‐min mark within the entire length of the recording was used for every participant for both PRE and POST. Recordings that had missing data, poor recording quality, or data/signal loss of more than 30% of the 5‐min segment were removed. Subsequently, an automated beat correction algorithm of Kubios software, which identifies and corrects missed, extra, and misaligned (including ectopic) beats from the recording was applied (Lipponen & Tarvainen, 2019).

**FIGURE 1:**
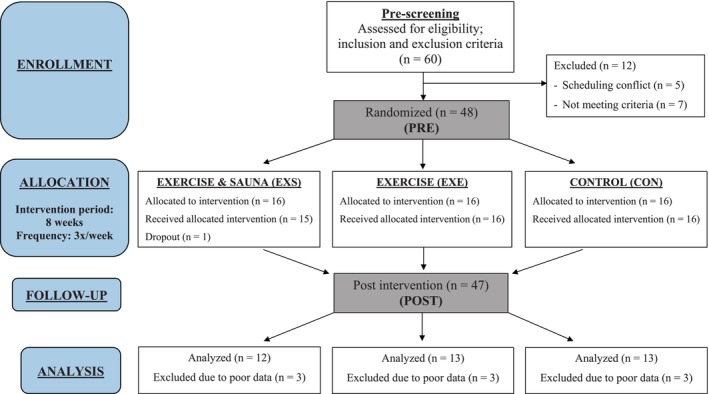
Experimental design (adapted and modified according to CONSORT guidelines template). n, number of human participants.

Recordings that presented a beat correction rate of over 5% were excluded from analysis. Nine sets of data were omitted as a result, leading to a final sample size of 38 (Figure 1). We also examined variations in participants' respiration rate for consistency, given that changes in respiratory patterns (i.e., change from high frequency range to low frequency range) are known to significantly affect HRV outcomes (Shaffer & Ginsberg, 2017). The Kubios software automatically removes slow nonstationary trends from the HRV data using a smoothness priors method (smoothing parameter: 500; cutoff frequency: 0.035 Hz), similar to a time‐varying high pass filter (Tarvainen et al., 2002).

The following time‐domain HRV variables were captured: mean RR interval (RR interval), root mean square of the successive differences between RR intervals (RMSSD), the standard deviation of the inter‐beat intervals for all normal sinus beats (SDNN), resting HR, and the average difference between the highest and lowest HRs during each analyzed 5‐min segment (HR_MAX_–HR_MIN_). The RMSSD represents the beat‐by‐beat variance in HR and is primarily used for the estimation of vagally‐mediated HRV changes (Shaffer et al., 2014). SDNN is a measure that expresses the fluctuations of all the factors contributing to HRV. Therefore, both parasympathetic and sympathetic nervous system activity influence it. It is also highly correlated to low frequency‐domain variables. In short‐term assessments, such as in the present study, the primary source of variation is respiratory sinus arrhythmia (RSA), which is mediated by parasympathetic activity (Shaffer & Ginsberg, 2017).

Frequency‐domain variables include high frequency power (HFP, 0.15–0.4 Hz) and low frequency power (LFP, 0.04–0.15 Hz). Frequency‐domain measurements can be expressed in absolute or relative power. In this study, both LFP and HFP are expressed in absolute units (ms^2^), calculated as ms squared divided by cycles per second (ms^2^/Hz). HFP represents parasympathetic or vagal activity and is often referred to as the respiratory band as it corresponds to RSA. Inspiration accelerates HR while expiration slows it. This modulation helps maintain the autonomic regulation necessary for cardiovascular health. LFP may be produced by a variety of sources. Primarily by the parasympathetic nervous system, by both the parasympathetic and sympathetic nervous system, the baroreceptors during BP regulation, or by baroreflex activity independently. However, during resting conditions LFP represents baroreflex activity and not cardiac sympathetic innervation (Shaffer & Ginsberg, 2017). Respiratory rate (RESP) was computed from RR interval data using an algorithm provided by the Kubios HRV software, which extracts respiration‐induced oscillations in the RR intervals, reflecting the cyclic variations in heart rate associated with the phases of the respiratory cycle.

### 2.4 Statistical analyses

To ensure that the statistical analyses were non‐biased, the data were analyzed separately by an independent statistician, who was blinded to the assignment and completely uninvolved in the research design, participant recruitment, and data collection processes. Prior to the intervention, the distributions of RR interval, HR_MAX_–HR_MIN_, and RESP were sufficiently close to being normally distributed to pass the Shapiro–Wilk test for normality, while all the other HRV variables failed it. This was also reflected in the absolute changes used here as response variables. The within‐group changes in RMSSD, SDNN, HR_MAX_–HR_MIN_ passed the normality test, while the changes in other variables were non‐normally distributed. The deviations observed here were mostly due to the presence of outliers, which are known to diminish the statistical power of tests based on assumptions of normality (Sprent & Smeeton, 2007). Thus, due to the presence of these outliers, we chose to perform the between‐group comparisons using the Mann–Whitney *U* test which is based on ranks. This allowed us to have 95.5% of the statistical power of the *t*‐test under perfect normality assumptions, while being robust against outliers (Sprent & Smeeton, 2007).

The experiment was originally designed with an effect size (Cohen's D) of 1 in mind (Lee et al., 2022), hence 16 individuals were assigned to each group, providing a statistical power of 80% for a two‐sample *t*‐test with a two‐tailed alternative hypothesis for normally distributed variables. However, our present interest is one‐way, that is, whether the EXE was a strong enough intervention to produce changes compared to CON, and whether EXS could produce additional effects compared to EXE. The statistical power of the setup was estimated by simulation, where for a given effect size, 100,000 normally distributed data sets were created. A statistical power of 80% could be achieved for an effect size of 1.06 with the final number of observations (*n* = 13 + 13 + 12 = 38). Thus, there is adequate statistical power and a high likelihood to detect differences of the magnitude that the experiment was designed for. It should be noted that the statistical power is based on simulations with the nonparametric Mann–Whitney tests, and thus ought to be robust against any differences in standard deviation and form of distribution in the population, as it only assumes that the ranks of the individuals follow a similar pattern. However, if effect sizes much below unity (e.g., 0.5 or 0.8) would be considered clinically significant, the statistical power will be lower and thus a type II error would be likely.

Statistical significance of mean differences between the groups in age, body mass, BMI, systolic BP, or the outcome variables prior to intervention (PRE) was tested using the Kruskal–Wallis test (one‐way ANOVA of ranks). As a sensitivity analysis, the Mann–Whitney comparisons were also repeated using a corresponding two‐sample *t*‐test and the Kruskal–Wallis comparisons using ANOVA.

For completeness, we also fitted linear mixed‐effects models with age, hypertension (SBP > 140), obesity (BMI ≥ 30), intervention group (CON, EXE, and EXS), timepoint (PRE and POST), and the interaction of group and timepoint as fixed effects. The models also included a random intercept term for individual. Further details and estimates, 95% confidence intervals, and *p* values are provided in the Tables S1–S6. Pearson's correlation between changes in maximal oxygen consumption (VO_2_max), which was found to differ between EXE and EXS in our previous work (Lee et al., 2022), and changes in the 10 response variables investigated in the present study are also reported therein. The statistical analyses were carried out using the statistical software R (R Core Team, 2024).

## 3 RESULTS

**FIGURE 2:**
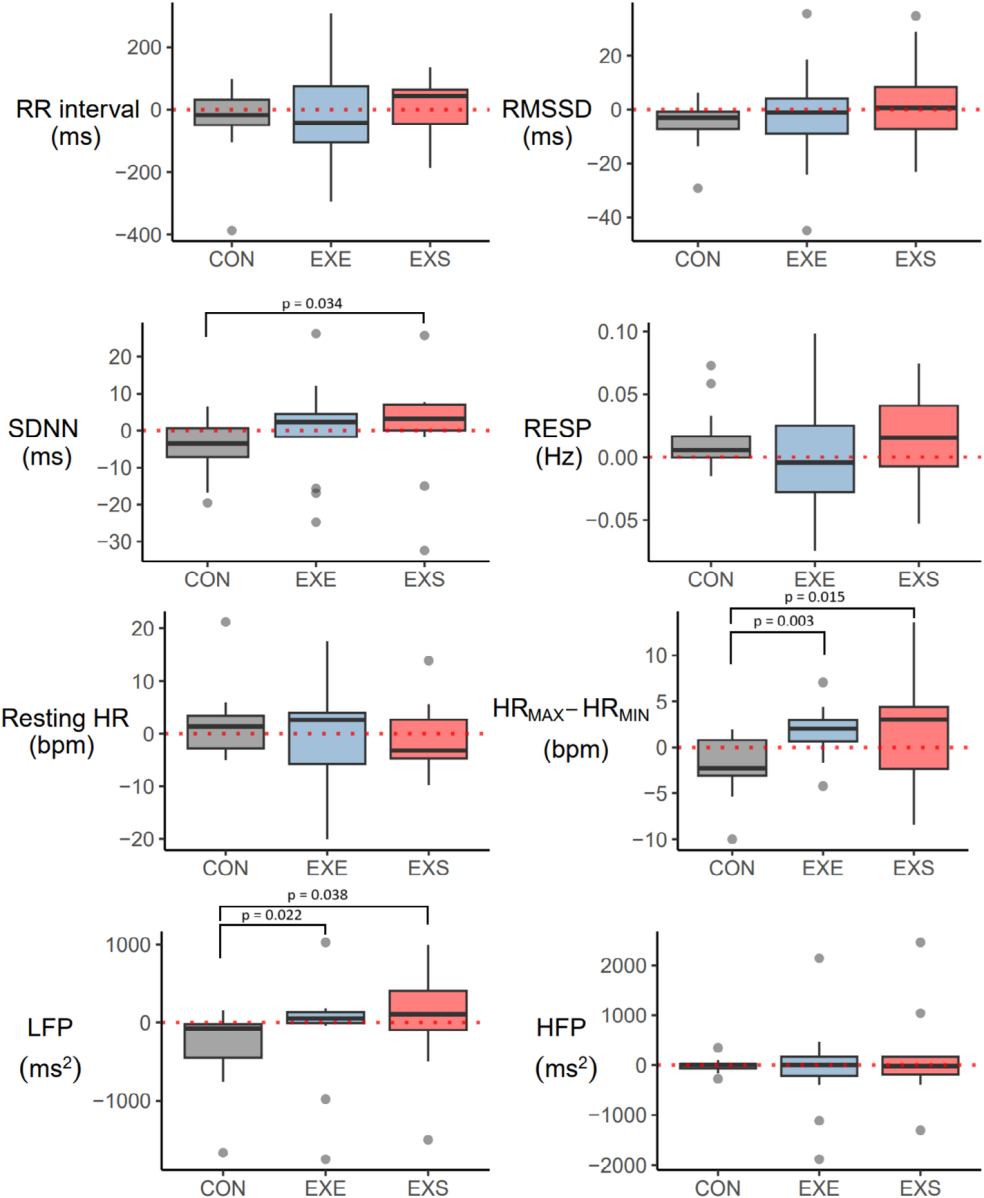
PRE‐POST change in HRV variables for control (CON), exercise (EXE), and exercise + sauna (EXS) groups. HFP, high frequency power; LFP, low frequency power; RESP, respiratory rate; RMSSD, root mean square of the successive differences between RR intervals; RR interval, time between successive R waves; SDNN, standard deviation of the inter‐beat intervals for all normal sinus beats. Box plot (s) represent the lower and upper quartiles, the line in the box denotes the median value. Minimum and maximum values are reflected by the whiskers. Gray dots are outliers, defined as values more than 1.5 times from the boxes' edge.

The characteristics of the participants are shown in Table 1. The three most commonly present traditional CVD risk factors were obesity (57.9%), elevated BP (36.8%), and family history of CHD (36.8%). No statistically significant differences between the groups were found for any of the variables in Table 1 (*p* = 0.081–0.993) or at PRE for the response variables (*p* = 0.189–0.989). The means of the HRV parameters and their standard deviations or interquartile ranges are presented in Table 2. The differences in HRV indices between EXE and CON are displayed in Table 3, and the comparison between EXS and EXE is displayed in Table 4. The changes in HRV variables are presented graphically for each group in Figure 2. Additional comparisons of RESP can be found in Tables S1 and S2. Age had an effect on RMSSD (Table S3), HFP (Table S4), and HR_MAX_–HR_MIN_ (Table S5). A moderately positive correlation was found between change in HR_MAX_–HR_MIN_ and change in VO_2_max, *r* (36) = 0.44, *p =* 0.007. A moderately inverse relationship was found between change in HR_MIN_ and VO_2_max, *r* (36) = −0.38, *p =* 0.02 (Table S6).

**TABLE 1:** Baseline characteristics of the participants.

Characteristics	Mean ± SD			
	Total *N* = 38 (males = 6)	Control (CON) *n* = 13 (*m* = 2)	Exercise (EXE) *n* = 13 (*m* = 2)	Exercise + sauna (EXS) *n* = 12 (*m* = 2)
Age (years)	49 ± 9	52 ± 6	50 ± 10	46 ± 9
Systolic BP (mmHg)	132 ± 16	131 ± 11	131 ± 13	136 ± 23
Diastolic BP (mmHg)	79 ± 10	81 ± 8	78 ± 10	79 ± 14
Body mass (kg)	89.8 ± 14.4	84.7 ± 15.2	89.6 ± 12.9	95.4 ± 14.2
Body height (m)	1.68 ± 0.08	1.66 ± 0.06	1.68 ± 0.07	1.72 ± 0.11
BMI (kg/m^2^)	31.8 ± 4.3	30.9 ± 4.9	32.1 ± 4.2	32.6 ± 3.8
Total cholesterol levels (mmol/L)	5.34 ± 0.93	5.57 ± 0.95	5.22 ± 0.97	5.21 ± 0.91
Risk factors^a^	Number (Percentage)	Number/Group		
Obesity (BMI > 30 kg/m^2^)	22 (57.9%)	7/13	8/13	7/12
Family history of CHD	14 (36.8%)	5/13	5/13	4/12
Elevated BP	14 (36.8%)	6/13	5/13	3/12
Elevated cholesterol (>6.2 mmol/L)	9 (23.7%)	4/13	3/13	2/12
Smoker (History of smoking)	5 (13.2%)	1/13	1/13	3/12

Abbreviations: BMI, body mass index; BP, brachial blood pressure; CHD, coronary heart disease; *m*, number of male participants; SD, standard deviation.

^a^ Number of participants with one, two, and three risk factors was 17, 18, and 4, respectively. No participant had more than three risk factors.

**TABLE 2:** PRE and POST measurements of the response variables for each group. The normally distributed variables are given with means and standard deviations, while the non‐normally distributed variables are given with means and interquartile ranges.

	Mean ± SD					
	Control (CON)		Exercise (EXE)		Exercise + Sauna (EXS)	
Normally distributed	PRE	POST	PRE	POST	PRE	POST
RR interval (ms)	970 ± 148	936 ± 119	1038 ± 184	1007 ± 142	939 ± 109	949 ± 108
HR_MAX_–HR_MIN_ (bpm)	11 ± 4	9 ± 3	10 ± 4	12 ± 4	11 ± 5	13 ± 5
RESP (Hz)^a^	0.22 ± 0.04	0.23 ± 0.03	0.24 ± 0.04	0.24 ± 0.04	0.22 ± 0.04	0.24 ± 0.06
Non‐normally distributed	Mean ± IQR					
Resting HR (bpm)	63 ± 17	65 ± 11	60 ± 12	61 ± 13	65 ± 12	64 ± 8
RMSSD (ms)	28 ± 14	24 ± 13	39 ± 15	36 ± 27	34 ± 25	37 ± 28
SDNN (ms)	28 ± 14	25 ± 9	34 ± 10	34 ± 20	34 ± 24	35 ± 32
LFP (ms^2^)	575 ± 771	284 ± 176	571 ± 511	495 ± 439	624 ± 807	670 ± 712
HFP (ms^2^)	350 ± 381	345 ± 478	776 ± 266	720 ± 547	659 ± 597	794 ± 721

Abbreviations: HFP, high frequency power; IQR, interquartile range; LFP, low frequency power; RESP, respiration rate; RMSSD, root mean square of the successive differences between RR intervals; SD, standard deviation; SDNN, standard deviation of the inter‐beat intervals for all sinus beats.

^a^ Secondary response variable where there were no between or within group differences (see Tables S2 and S3).

**TABLE 3:** Changes in HRV variables in the control and exercise groups, their 95% confidence intervals, *p* values for differences in group means (Mann–Whitney), and the direction of the alternative hypotheses.

	Control (CON) *n* = 13 (*m* = 2)		Exercise (EXE) *n* = 13 (*m* = 2)		*p*‐Value	Alternative hypothesis
	Mean change (POST‐PRE)	95% CI	Mean change (POST‐PRE)	95% CI		
HR_MAX_–HR_MIN_ (bpm)	−2	−4, 0	2	0, 3	0.003**	CON<EXE
Resting HR (bpm)	2	−2, 6	1	−5, 8	0.56	CON<EXE
RR interval (ms)	−34	−107, 38	−32	−136, 74	0.62	CON<EXE
RMSSD (ms)	−4	−10, 2	−2	−14, 10	0.46	EXE<CON
SDNN (ms)	−4	−8, 1	0	−8, 8	0.13	CON<EXE
LFP (ms^2^)	−291	−590, 7	−76	−470, 317	0.022*	CON<EXE
HFP (ms^2^)	−5	−95, 84	−57	−610, 497	0.64	CON<EXE

Abbreviations: CI, confidence intervals; HFP, high frequency power; HR, heart rate; *m*, number of male participants; RMSSD, root mean square of the successive differences between RR intervals; SDNN, standard deviation of the inter‐beat intervals for all normal sinus beats; LFP, low frequency power.

*p < 0.05, **p < 0.01.

**TABLE 4:** Changes in HRV variables in the exercise and exercise + sauna groups, their 95% confidence intervals, *p* values for differences in group means (Mann–Whitney), and the direction of the alternative hypotheses.

	Exercise + sauna (EXS) *n* = 12 (*m* = 2)		Exercise (EXE) *n* = 13 (*m* = 2)		*p* Value	Alternative hypothesis
	Mean change (POST‐PRE)	95% CI	Mean change (POST‐PRE)	95% CI		
HR_MAX_–HR_MIN_ (bpm)	1	−3, 6	2	0, 3	0.45	EXE<EXS
Resting HR (bpm)	−1	−5, 3	1	−5, 8	0.73	EXE<EXS
RR interval (ms)	10	−48, 67	−32	−136, 74	0.23	EXE<EXS
RMSSD (ms)	3	−8.0, 13.5	−2	−14, 10	0.71	EXE>EXS
SDNN (ms)	1	−8, 10	0	−8, 8	0.29	EXE<EXS
LFP (ms^2^)	46	−359, 451	−76	−470, 317	0.23	EXE<EXS
HFP (ms^2^)	135	−438, 707	−57	−610, 497	0.41	EXE<EXS

Abbreviations: CI, confidence intervals; HFP, high frequency power; HR, heart rate; LFP, low frequency power; *m*, number of male participants; RMSSD, root mean square of the successive differences between RR intervals; SDNN, standard deviation of the inter‐beat intervals for all normal sinus beats.

### 3.1 Exercise (EXE) versus control (CON)

Average PRE‐POST differences were found between the CON and EXE groups for HR_MAX_–HR_MIN_ (~4 bpm, *p* = 0.003) and LFP (215 ms^2^, *p* = 0.022). Specifically, HR_MAX_–HR_MIN_ was increased for the EXE group compared to the CON group (Table 3). Unexpectedly, the LFP values for the CON group appeared to decrease, while the EXE group remained approximately the same. In the sensitivity analysis, the conclusions were similar for HR_MAX_–HR_MIN_, but the LFP results were not statistically significant (*p* = 0.177), reflecting the effect of the outliers shown in Figure 2. The difference in HR_MAX_–HR_MIN_ change was also statistically significant and of a similar size in the linear mixed‐effects model. The LFP difference was in the same direction but not statistically significant.

### 3.2 Exercise and sauna (EXS) versus exercise (EXE)

There were no significant differences found in any of the captured HRV indices between EXS and EXE (Table 4). This result was also indicated by the sensitivity analysis. No statistically significant differences were found with the linear mixed‐effects model either.

## 4 DISCUSSION

We investigated the effects of regular exercise and the combination of exercise and sauna bathing on HRV in middle‐aged adults with CVD risk. The main findings were significant differences in the change in HR_MAX_–HR_MIN_ and LFP between the EXE and CON groups following the 8‐week intervention. Specifically, HR_MAX_–HR_MIN_ increased in the EXE group, while LFP showed a decrease in the CON group. However, there were no significant differences in any of the HRV indices between the EXE and EXS groups. These findings suggest that regular exercise according to the recommended guidelines can elicit improvements to cardiac autonomic function among individuals with elevated CVD risk. However, the incorporation of regular sauna bathing postexercise does not appear to enhance these benefits.

### 4.1 Effects of exercise on time‐domain measures and HR

Regular exercise has been shown to increase HRV in a similar population (Earnest et al., 2008; Jurca et al., 2004). Regular aerobic exercise leads to an increase in parasympathetic activity (Grässler et al., 2021), and those with CVD risk benefit from lowered sympathetic nervous system adaptations as well (Carter & Ray, 2015). Our results show that 8 weeks of regular exercise performed three times a week did not significantly change resting HR or the time‐domain measure of RMSSD, which is a measure of parasympathetic activity that has a relationship with vagal modulation (Malik et al., 1996; Shaffer & Ginsberg, 2017). This is in contrast to the results of an earlier study with a similar intervention (Jurca et al., 2004), which showed reduced resting HR and an increase in RMSSD.

Indeed, it has been postulated previously (Sandercock et al., 2005) that changes in resting HR can occur in as quickly as 4 weeks of aerobic exercise. However, more recent evidence (Reimers et al., 2018) suggests that resting HR adaptations occur only after 12 weeks of three sessions per week of aerobic exercise. This is in line with the results from the present study: RR interval, RMSSD, SDNN, and resting HR showed no change after the 8‐week intervention. Obesity may also have been a factor. It has previously been shown that a 10% increase in body weight is associated with a decrease in parasympathetic activity and an increase in average resting HR (Hirsch et al., 1991). As such, obesity as a factor cannot be ruled out, given that the majority of the participants in the present study were classified as obese. Furthermore, the EXE group did indeed lower their fat mass after the intervention compared to the CON group (Lee et al., 2022), which supports this postulation.

It has also been well documented that the magnitude of effects on HRV depends on factors like age, exercise intensity and/or frequency, and intervention length (Grässler et al., 2021). As such, it is likely that these factors played a role in the lack of change seen in the time‐domain measurements of the present study. However, HR_MAX_–HR_MIN_ did show improvement after our 8‐week intervention. HR_MAX_–HR_MIN_ in the present study represents the highest‐to‐lowest variation in HR within each analyzed 5‐min segment, and while it does not directly reflect autonomic nervous system modulation, it serves as an indicator of overall cardiovascular function. Nevertheless, an increase in HR_MAX_–HR_MIN_, while not a traditional HRV measure, suggests an enhanced capacity for heart rate modulation connected to RSA (Grossman & Taylor, 2007). RSA is the natural increase in HR during inspiration and the decrease during expiration, primarily influenced by the parasympathetic nervous system and baroreflex mechanisms (Karemaker, 2009). This could reflect improvements in cardiovascular dynamics, potentially influenced by underlying autonomic mechanisms without direct measurement. However, a deep breathing test is required to determine if RSA mechanisms provoke a greater HR_MAX_–HR_MIN_.

HR modulation during respiratory cycles reflects the RSA; accelerating during inspiration and decelerating during expiration via vagus nerve activity (Garcia et al., 2013). Indeed, recent studies have also used HR_MAX_–HR_MIN_ as a surrogate measure of RSA (Meehan & Shaffer, 2023; Shaffer et al., 2022), and higher levels of physical activity were associated with higher RSA in a similar cohort of middle‐aged European adults (Hu et al., 2017). In addition, to our best knowledge, this is the first exercise‐based interventional trial that reported changes in RSA, represented by HR_MAX_–HR_MIN_ in a group of middle‐aged adults with CVD risk factors. However, because the data was not collected under controlled respiratory conditions, this must be interpreted with caution. Moreover, it is important to note that HR_MAX_–HR_MIN_ is not truly representative of RSA. Nevertheless, we found a positive relationship between change in HR_MAX_–HR_MIN_ and change in VO_2_max using data from our previous study (Lee et al., 2022). Improvements in VO_2_max are typically driven by improvements in HR; the ability to achieve a higher HR increases cardiac output and consequently VO_2_max. Additionally, change in HR_MIN_ was negatively correlated to VO_2_max, which suggests that improvements in cardiorespiratory fitness were associated with reductions in HR.

### 4.2 Effects of exercise on frequency domain measures

Our results showed a decrease in LFP in the CON group compared to EXE group. However, we found no significant changes in HFP. Several ways of interpreting LFP during supine rest have been put forth. Some have thought it to be a measure of sympathetic activity (Malliani et al.,  1991). Correlations between LFP and sympathetic activity (DeBeck et al., 2010) as well as the influence of vagal modulation on LFP have also been documented previously (Pagani et al., 1997). However, more up‐to‐date evidence has ruled out an overly simplistic, direct relationship between LFP and sympathetic activity. Instead, in resting conditions, LFP has been suggested to be a measure of modulation of cardiac autonomic outflows by baroreflexes (Berntson et al., 1997; Goldstein et al., 2011; Heathers, 2014; Malik et al., 1996; Shaffer & Ginsberg, 2017). Specifically, (Rahman et al., 2011) found that LFP has a strong positive correlation with baroreflex‐cardiovagal gain, and no correlation with cardiac sympathetic innervation. In addition, these findings, observed during both sitting and the Valsalva maneuver, indicate that patients with lower baroreflex sensitivity exhibited reduced LF power, independent of their cardiac sympathetic innervation status. In healthy adults, physically active individuals tend to have better baroreceptor function than those who are sedentary (Lester et al., 2022). As such, a decrease in LFP could be interpreted as a reduction in baroreceptor function in our study using a population with CVD risk factors. This is corroborated somewhat by a reduction in cardiorespiratory fitness for the CON group in the same cohort who maintained their sedentary behavior (Lee et al., 2022). However, it must be noted that we did not directly assess baroreflex sensitivity with the Valsalva maneuver test, which is one of the most widely used methods for this purpose (La Rovere et al., 2011). Therefore, this interpretation remains inferential in the context of the current data.

HRV has also been demonstrated to be sensitive in detecting the transition to menopause, postmenopause, and the intensity of menopause‐related symptoms (Martinelli et al., 2020). Given that the majority of the participants were middle‐aged women, the present HRV data may have been confounded by these factors. Indeed, the sex differences in HRV and LFP have been well‐established (Koenig & Thayer, 2016). Briefly, women tend to have lower LFP and show greater parasympathetic modulation of cardiovascular activity compared to men. Regular exercise has been documented to enhance parasympathetic activity throughout the phases of the menstrual cycle (Shetty et al., 2012), and increase HRV in postmenopausal women (Earnest et al., 2008; Jurca et al., 2004). Therefore, it is reasonable to postulate that regular exercise had protective effects against the decrease in LFP due to the CON group's sedentary behavior.

### 4.3 Effects of exercise and sauna bathing on HRV

We did not find differences in any of the HRV indices between EXE and EXS after the intervention. This is somewhat consistent with the first acute study exploring HRV in exercise and sauna (Gayda et al., 2012) that found no differences in frequency‐domain indices between a single session of aerobic exercise followed by sauna bathing, and sauna bathing alone. However, compared to controls, sauna bathing induced acute modulations to the cardiovascular system, via decreased parasympathetic and increased sympathetic control of the heart (Gayda et al., 2012). These findings suggest that sauna bathing may enhance cardiac autonomic nervous system activity when performed regularly. Indeed, a 6‐week sauna treatment program was able to improve autonomic nervous system function (Kunbootsri et al., 2013). However, the exposure duration (30 min) was twice the amount employed in the present study, using a study population of young Asians with a healthy BMI. The present study participants were predominantly obese or overweight middle‐aged individuals, which could have blunted the physiological adaptations and responses to the sauna intervention (Speakman, 2018). It is also likely that the cardiac autonomic adaptations to regular exercise may have overlapped and masked that of sauna bathing (Ferreira et al., 2023).

In a more recent study, HRV remained unchanged after 10 days of exercise and sauna bathing at higher temperatures and a longer duration (Leicht et al., 2018). The authors postulated that the total heat stress applied over the course of the intervention may have been insufficient for adaptations (Taylor, 2014). This cannot be ruled out, as the heat stress applied in the present study was 26,100 arbitrary units (duration in minutes × frequency in days × temperature of heat stress used in Celsius), which is nearly identical to what Leicht and associates (Leicht et al., 2018) used. Despite having sufficient statistical power to detect meaningful differences in our data, we found no differences in any of the captured HRV indices between EXE and EXS. Based on the current body of evidence, we believe that regular sauna bathing may have some effect on HRV, but it is unlikely to be a profound one.

Future studies should explore the effects of combining exercise and sauna bathing in a more diverse demographic, including younger and/or healthier individuals with varying levels of cardiovascular fitness. Finally, it is also essential to report and utilize higher heat loads/stress in sauna bathing and heat therapy studies, which would allow us to better understand thresholds and ceilings in responses and adaptations. This would help facilitate study comparisons and systematic reviews in this growing field.

### 4.4 Limitations

Several limitations from the present study need to be noted. Data from nine participants was omitted. As such, the modest resultant cohort size may limit the generalizability of our findings. However, this was done to preserve the integrity of the data set used. The analyzed data were based on short‐term recordings (5‐min) and are not directly comparable to measurements using long‐term recordings (≥24 h), which is considered to be more robust (Hayano & Yuda, 2021). However, short‐term recordings under controlled conditions may offer greater physiological specificity, especially in experimental settings such as ours. We did not control for respiration‐related confounders during our data collection, which we are aware may have had a bearing on the present results (Laborde et al., 2017). Nevertheless, we did not find statistically significant differences based on the software‐estimated respiration rates. The study lacked a sauna‐only group, which would have provided information on the HRV adaptations to regular sauna bathing, allowing us to ascertain if these adaptations were indeed masked by those of regular exercise. It also limits the mechanistic conclusions regarding sauna's role in autonomic modulation. However, that was not the main objective of the present experimental setup. Additionally, the wide confidence intervals observed in our results reflect substantial individual variability. This suggests that individual differences in response to the interventions could play a role in the outcomes measured. In addition, autonomic tests could be performed for a better physiological interpretation since some patterns are not observed at rest (Ferreira et al., 2021). While there were significant differences between the CON and EXE groups in the PRE‐POST differences of LFP, caution is warranted in attributing these to exercise alone. These differences are likely due to the decrease in the CON group rather than an increase in the EXE group per se. These changes may have been confounded by the menstrual cycle or menopause status of the participants, which were not controlled for. In particular, outcomes such as LFP and RMSSD are known to be strongly influenced by sex, menstrual cycle phase, and menopausal status (Koenig & Thayer, 2016; Martinelli et al., 2020). In addition, the present results need to be appropriately contextualized as there was a low number of male participants in each group. BMI could also have influenced these results.

## 5 CONCLUSION

Our investigation into the effects of regular exercise and adjunctive use of sauna bathing on HRV reveal salutary adaptations from regular exercise, validating the current physical activity guidelines. However, the anticipated additive benefits of sauna bathing on cardiac autonomic function, beyond those achieved through regular exercise, were not observed. These findings underscore the value of regular exercise a cornerstone for improving HRV in the middle‐aged population with CVD risk. The HRV adaptations to regular exercise may also have masked the effects of regular sauna bathing. Therefore, the role of regular sauna bathing as a standalone therapeutic modality still requires further investigation.

## AUTHOR CONTRIBUTIONS

EL and JPA conceived and designed research; EL, IK, and JPA performed experiments; JK analyzed data; EL, SK, PW, and HH interpreted results of experiments; JK prepared figures; EL, SK, PW, JK, HH, and EL drafted manuscript; EL, SK, PW, JK, IK, HH, and JPA edited and revised manuscript; EL, SK, PW, JK, IK, HH, and JPA approved final version of manuscript.

## FUNDING INFORMATION

The Finnish Cultural Foundation (SKR) partially funded the corresponding author's salary (Grant No.: 00190620 to EL).

## CONFLICT OF INTEREST STATEMENT

The authors report no conflict of interest.

## ETHICAL STATEMENT

Ethical approval was granted by the institutional review board of the Central Finland Hospital District ethical committee, Jyväskylä, Finland (Dnro 3 U/2019).

## Supporting information


Tables S1–S6.

## Data Availability

The data that support the findings of this study are available from the corresponding author, SK, upon reasonable request.
