# Supplementary material for: Regular postexercise sauna bathing does not improve heart rate variability: A multi‐arm randomized controlled trial
Source: Physiol Rep. 2025 Jul 3;13(13):e70449. doi: 10.14814/phy2.70449 (PMC12231185; doi:10.14814/phy2.70449)
Supplement: Supplementary file 1 — Tables S1–S6. [file PHY2-13-e70449-s001.docx]

**Supplementary Table 1.** Changes in HRV variables in the control and exercise groups, their 95% confidence intervals, p-values for differences in group means (Mann–Whitney), and the direction of the alternative hypotheses.

|  | **Control (CON)**  **n = 14 (m = 2)** | | **Exercise (EXE)**  **n = 13 (m = 2)** | | **p-value** | **Alternative hypothesis** |
| --- | --- | --- | --- | --- | --- | --- |
|  | **Mean change (POST-PRE)** | **95% CI** | **Mean change (POST-PRE)** | **95% CI** |  |  |
| **RESP (Hz)^a^** | 0.013 | -0.002, 0.030 | 0.017 | -0.006, 0.040 | 0.20 | CON≠EXE |
| **RESP (Hz)^b^** | 0.08 | -0.01, 0.17 | 0.02 | -0.10, 0.14 | 0.19 | CON≠EXE |
| Note: CI, confidence intervals; LFP, low frequency power; HFP, high frequency power; RESP, respiration rate. ^a^ absolute change, ^b^ relative change | | | | | | |

**Supplementary Table 2.** Changes in HRV variables in the control and exercise groups, their 95% confidence intervals, p-values for differences in group means (Mann–Whitney), and the direction of the alternative hypotheses.

|  | **Exercise + sauna (EXS)**  **n = 12 (m = 2)** | | **Exercise (EXE)**  **n = 13 (m = 2)** | | **p-value** | **Alternative hypothesis** |
| --- | --- | --- | --- | --- | --- | --- |
|  | **Mean change (POST-PRE)** | **95% CI** | **Mean change (POST-PRE)** | **95% CI** |  |  |
| **RESP (Hz)^a^** | 0.017 | -0.006, 0.040 | -0.002, 0.030 | -0.028, 0.028 | 0.27 | EXE≠EXS |
| **RESP (Hz)^b^** | 0.08 | -0.02, 0.18 | 0.02 | -0.10, 0.14 | 0.35 | EXE≠EXS |
| Note: CI, confidence intervals; LFP, low frequency power; HFP, high frequency power; RESP, respiration rate.  ^a^ absolute change, ^b^ relative change | | | | | | |

**Supplementary Table 3.** Fixed effects for log-transformed RMSSD (ms)

|  | Estimate | SE | *p*-value |
| --- | --- | --- | --- |
| Intercept *(*ref. *EXE. PRE)* | 4.71 | 0.59 | <0.0001* |
| *EXS* | -0.21 | 0.24 | 0.38 |
| *CON* | -0.27 | 0.24 | 0.27 |
| Time (*POST*) | -0.05 | 0.13 | 0.69 |
| Age | -0.02 | 0.01 | 0.04* |
| BMI ≥ 30 | 0.00 | 0.18 | 0.99 |
| SBP > 140 | -0.27 | 0.22 | 0.23 |
| *EXS×POST* | 0.02 | 0.19 | 0.92 |
| *CON×POST* | -0.08 | 0.19 | 0.67 |

**Supplementary Table 4.** Fixed effects for log-transformed HFP

|  | Estimate | SE | *p*-value |
| --- | --- | --- | --- |
| Intercept *(*ref. *EXE. PRE)* | 8.36 | 1.24 | <0.0001* |
| *EXS* | -0.26 | 0.50 | 0.61 |
| *CON* | -0.40 | 0.50 | 0.43 |
| Time (*POST*) | -0.07 | 0.28 | 0.80 |
| Age | -0.05 | 0.02 | 0.04* |
| BMI ≥ 30 | 0.09 | 0.39 | 0.82 |
| SBP > 140 | -0.51 | 0.47 | 0.29 |
| *EXS×POST* | -0.14 | 0.40 | 0.72 |
| *CON×POST* | -0.03 | 0.39 | 0.93 |

**Supplementary Table 5.** Fixed effects for log-transformed HFP Fixed effects for HR_MAX_ - HR_MIN_ (bpm)

|  | Estimate | SE | *p*-value |
| --- | --- | --- | --- |
| Intercept *(*ref. *EXE. PRE)* | 18.79 | 3.76 | <0.0001* |
| *EXS* | 0.62 | 1.65 | 0.71 |
| *CON* | 1.22 | 1.64 | 0.46 |
| Time (*POST*) | 1.69 | 1.23 | 0.18 |
| Age | -0.18 | 0.07 | 0.02* |
| BMI ≥ 30 | 0.51 | 1.18 | 0.67 |
| SBP > 140 | -0.62 | 1.41 | 0.66 |
| *EXS×POST* | -0.27 | 1.77 | 0.88 |
| *CON×POST* | -3.65 | 1.74 | 0.04* |

**Supplementary Table 6.** Correlation between change in VO_2MAX_ and change in study variables

| **Variable** | **Correlation** | **95% C.I.** | **p-value** |
| --- | --- | --- | --- |
| Mean RR (ms) | 0.29 | [-0.04, 0.57] | 0.08 |
| SDNN (ms) | 0.27 | [-0.07, 0.55] | 0.11 |
| Mean HR (bpm) | -0.29 | [-0.57, 0.04] | 0.09 |
| Min HR (bpm) | -0.38 | [-0.63, -0.06] | 0.02* |
| Max HR (bpm) | -0.09 | [-0.40, 0.25] | 0.62 |
| RMSSD (ms) | 0.24 | [-0.10, 0.53] | 0.16 |
| LFpow_FFT (ms2) | 0.29 | [-0.04, 0.56] | 0.09 |
| HFpow_FFT (ms2) | 0.07 | [-0.27, 0.39] | 0.71 |
| RESP (Hz) | 0.10 | [-0.24, 0.41] | 0.58 |
| HR_MAX_ - HR_MIN_ (bpm) | 0.44 | [0.14, 0.67] | 0.007* |
